# Disturbances in primary dental enamel in Polish autistic children

**DOI:** 10.1038/s41598-020-69642-3

**Published:** 2020-07-29

**Authors:** Marta Kurek, Beata Borowska, Beata Lubowiedzka-Gontarek, Iwona Rosset, Elżbieta Żądzińska

**Affiliations:** 1 Department of Anthropology, Faculty of Biology and Environmental Protection, University of Łódź, Banacha 12/16, 90-237 Łódź, Poland; 2 Department of Pedodontics, Dental Institute in Łódź, Teaching Hospital No. 6, 92-213 Łódź, Poland; 3 Visiting Research Fellow in the School of Medical Sciences, Faculty of Health Sciences, The University of Adelaide, South Australia, 5005 Australia

**Keywords:** Autism spectrum disorders, Oral anatomy

## Abstract

Dental enamel is a structure that is formed as a result of the regular functioning of ameloblasts. The knowledge of the patterns of enamel secretion allows an analysis of their disruptions manifested in pronounced additional accentuated lines. These lines represent a physiological response to stress experienced during enamel development. The aim of this study was to assess the occurrence of accentuated lines in the tooth enamel of autistic boys. The width of the neonatal line and the periodicity of the striae of Retzius were also assessed. The study material consisted of longitudinal ground sections of 56 primary teeth (incisors and molars): 22 teeth from autistic children and 34 teeth from the control group. The Mann–Whitney U test indicates that the accentuated lines were found significantly more often in autistic children (Z = 3.03; *p* = 0.002). No differentiation in the rate of enamel formation and in the rate of regaining homeostasis after childbirth were found. The obtained results may indicate a higher sensitivity of autistic children to stress factors, manifested in more frequent disturbances in the functioning of ameloblasts or may be a reflection of differences in the occurrence of stress factors in the first years of life in both analyzed groups.

## Introduction

Autistic patients are characterized by developmental disorders and specific age-dependent effects^1^. Although autism spectrum disorders (ASDs) are genetic in origin, it is known that a variety of environmental preconceptions and prenatal influences also plays a critical role in their emergence and in their subsequent course^2,3^. There is a lack of publications dealing with dental disturbances in autistic children. However, previous research has shown that in cognitive and motor delayed children with ASD-related gene mutation (activity-dependent neuroprotective protein gene—ADNP), premature primary tooth eruption is observed^4^. In this study, 44 out of 54 ADNP-mutated children (81%) have almost fully erupted teeth, including molars, by 1 year of age.

Dental enamel is a structure that is formed as a result of the regular secretion by ameloblasts, visible in the form of cross-striations reflecting the circadian rhythm of the functioning of ameloblasts and of lines or bands (striae of Retzius) following an approximately weekly rhythm^5–9^. Knowledge of enamel secretion patterns of the various tooth types^10–15^ allows an analysis of their disruptions manifested in pronounced additional accentuated lines^7,15^. These lines are indicative of mineralization disturbances and of a slower rate of enamel formation by the ameloblasts present in the enamel-forming front at a given time, and they represent a physiological response to the stress experienced during enamel development^16–19^. In the case of primary teeth, enamel formation begins on average 189 or 176 days before birth and ends about 396 days after birth^20–22^. This constitutes a record of stressful events experienced by an individual up to approx. 1.2 years of life.

According to the literature, the factors which may give rise to accentuated lines include: maternal infections during pregnancy^15^, childhood diseases^23^, immunization/vaccination in the first year of life^15^, resource seasonality and periods of drought in the case of non-human primates^24,25^, as well as undernourishment or dietary transitions, such as weaning^26^. A higher number of accentuated lines in the enamel of primary second molars has also been observed in children with developmental disturbances caused by genetic defects, such as familial dysautonomia syndrome (hereditary sensory and autonomic neuropathy)^27^.

A specific kind of an accentuated line is the neonatal line (NNL). It is observed in all deciduous teeth and is formed during the perinatal period. It separates the enamel formed prenatally from that formed postnatally, and its width is connected with perinatal factors, including the duration of delivery and the type of delivery, with the intake of certain medicines by the mother or with the child’s season of birth^10,28–30^.

The exact causes of autism are not fully understood. Numerous studies into the etiology of ASD, largely motivated by its increasing incidence in Europe and in the USA, point both to genetic defects^31^ and to environmental factors, such as viral infections, metabolic imbalances, and exposure to noxious chemicals during pregnancy^32^. It also appears that some children are born with a susceptibility to autism, but researchers have not yet identified a single trigger that causes autism to develop. Autism tends to occur more frequently than expected among individuals who have certain medical conditions, including fragile X syndrome^33^, tuberous sclerosis^34^, congenital rubella^35^, and untreated phenylketonuria^36^. Some harmful substances, such as ethanol, valproic acid, and misoprostol ingested during pregnancy have also been associated with an increased risk of autism^37^.

Thus, it may be assumed that early ontogenetic disruptions in autistic children are also reflected in additional, irregular accentuated lines in dental enamel.

The aim of this study was to assess the occurrence of accentuated lines in the primary teeth of Polish autistic children. To the best of our knowledge, this is the first paper dealing with this issue.

## Material and methods

### Material

The study material consisted of primary teeth free from any developmental defects or dental caries. A total of 56 teeth were analyzed: 22 primary teeth from autistic boys (17 incisors including 15 i^1^, 2 i_2_ and 5 molars including 3 m^1^, 1 m_1_, 1 m_2_) and 34 teeth from the control group of boys (25 incisors including 15 i^1^, 7 i^2^, 3 i_2_ and 9 molars including 2 m^1^, 1 m^2^, 2 m_1_, 4 m_2_). In the study, one tooth came from one child. In the case of the control subjects, teeth were sampled from children aged 5 to 10 years attending kindergartens and primary schools in Łódź (a city in central Poland with approximately 700,000 inhabitants) and from volunteers in a program called “tooth fairy”. Odontological material from autistic children was obtained at the Institute of Dentistry, Central Teaching Hospital of the Medical University in Łódź. All extractions were performed for orthodontic reasons (when deciduous teeth that could disturb the process of dentition were still present in the oral cavity, although permanent teeth were already erupting) or during routine dental check-ups, when a deciduous tooth that would be shed in a moment was gently removed with the parents’ consent. The teeth from the autistic children were also collected in kindergartens and in primary schools in Łódź for children with disabilities. All the autistic children were diagnosed with autism spectrum disorders. All procedures were carried out in accordance with the relevant regulations including obtaining informed consent from the parents of the children whose teeth were collected for enamel analysis. All experimental protocols were approved by the Ethical Commission at the University of Łódź (No. KBBN-UL/II/9/2010).

All the children were born between the 37^th^ and the 42^nd^ week of gestation (full-term). The average birth parameters fell into the range typical of full-term newborns in Łódź^38^ and were as follows: for the control children: mean body weight = 3,374.7 g, SD = 393.0 g, and mean body length = 54.6 cm, SD = 2.6 cm; for the autistic children: mean body weight = 3,485.9 g, SD = 571.5 g, and mean body length = 55.2 cm, SD = 2.6 cm. There was no statistically significant difference in body weight (Z = 0.64; *p* = 0.52) and in body length (Z = 0.87; *p* = 0.38) between the healthy and the autistic children whose teeth were analyzed.

Each tooth was cleaned in a bath containing 70% alcohol for 24 h and dried with compressed oil-free air. Sections of the sampled teeth were made using a 0.5 mm diamond-wafering blade (Buehler IsoMet 1,000), followed by specimen embedding in epoxy resin (Biodur). The teeth were sectioned along the long axis in the labiolingual plane. The sections passed through the tips of the dentine horns and the tips of the enamel cusps. In order to secure an “ideal plane” of the section coinciding most precisely with the long axis of the tooth (a strictly radial plane), the cut made with the diamond saw was slightly shifted in the distal or medial direction. Subsequently, excess material was removed using abrasive paper (grades 600, 1,000, and 2,400). This procedure minimized the obliquity of the sectioned specimens^20,21^.

For each section, series of photomicrographs were taken with a Delta Optical HDCE—50B camera attached to a light microscope (Delta Optical Evolution 300) with an apochromatic objective lens 10 × /0.65 ∞/0.17. The images were used for an assessment of the number of accentuated lines. During the analysis of the images, the procedure described in the studies by^8,25,26^ was used, according to which clearly marked lines standing out in the structure of the enamel and visible through 75% of their length from the EDJ to the surface of the tooth were recognized as accentuated lines.

The width of the neonatal line was estimated for all the examined teeth. The measurement was performed in two places on the crown of the tooth on the labial surface, along the course of the enamel prisms. In the case of the incisors, the measurements were performed in the central part of the crown and in the proximity of the dentine horn. In the case of the molars, the places of measurement were located in the proximity of the dentine horn of both cusps and in the area between them.

The distances between the striae of Retzius were assessed in the above-mentioned parts of the crown. The values concerning the rate of enamel formation were obtained on the basis of measurements between three pairs of adjacent striae of Retzius. The assessment was performed in the central part of the postnatal enamel, along the course of the enamel prisms, from the edge of the Retzius line closest to the enamel-dentin junction to the edge of the next line. The measurements were subsequently used in the regression formula proposed by^15^. The method proposed by these authors allows determining the mean time of enamel formation in days by using data about the length of the enamel prisms. It also served to estimate the time of formation of accentuated lines in the enamel of the analyzed teeth. Measurements were performed of the distance from the neonatal line to the individual accentuated lines visible in the enamel, and the obtained values were substituted into the formula.

All the above-mentioned measurements were performed twice in order to minimize the measurement error, and on their basis, mean values were calculated.

### Statistical analysis

The Mann–Whitney U test was used to examine differences between the mean numbers of disruptions in deciduous enamel, the average width of the neonatal line, and the periodicity of Retzius lines observed in autistic and in healthy boys.

All statistical analyses were performed using STATISTICA 12.0 software.

## Results

**Figure 1:**
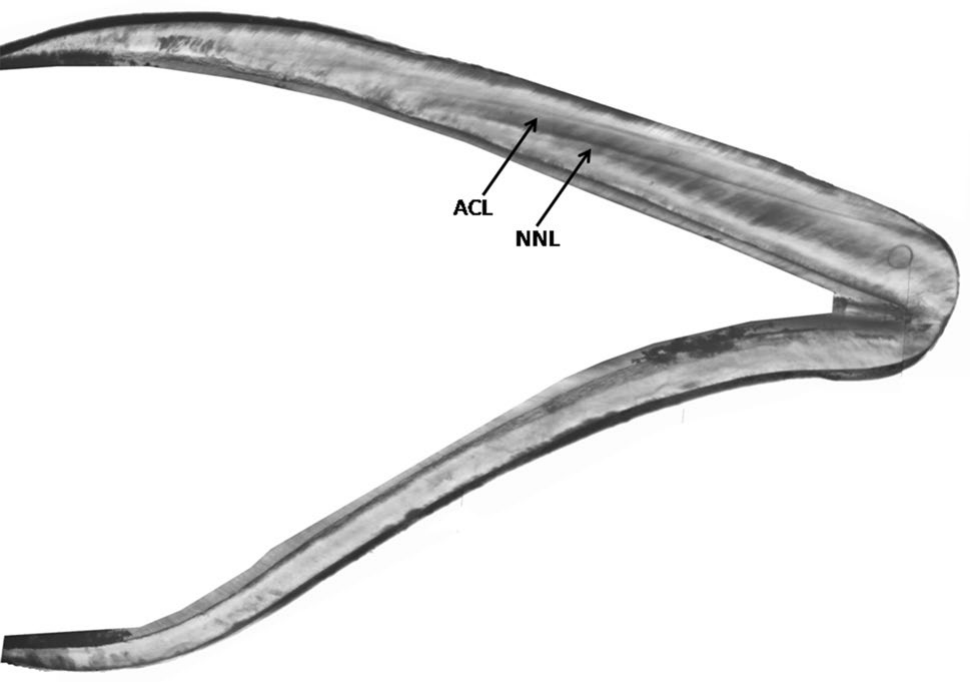
The enamel of maxillary second deciduous incisor of autistic boy with visible neonatal line (NNL) and one accentuated line (ACL) (magnification 4x).

**Figure 2:**
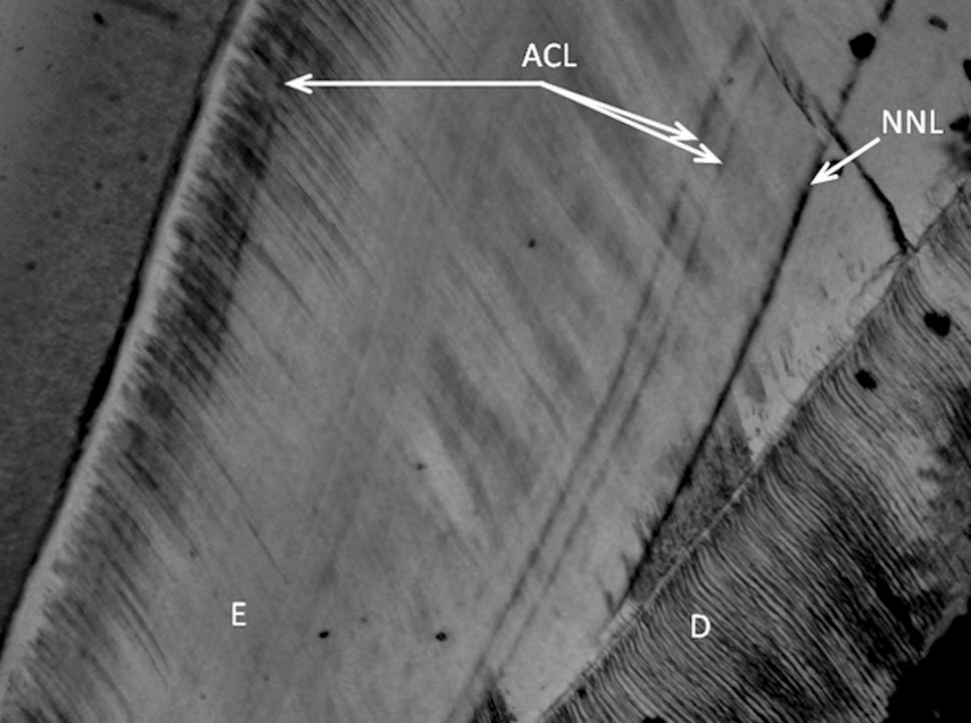
Accentuated lines in enamel of healthy children (ACL—accentuated lines, NNL—neonatal line, E—enamel, D—dentin) (magnification 40x).

Accentuated lines in dental enamel were found significantly more often in autistic children (Z = 3.03; *p* = 0.002) (Table 1). Figure 1 shows the enamel of incisor with one accentuated line. The enamel of the maxillary first molar of a healthy boy with three visible accentuated lines is presented in Fig. 2.

**Table 1:** Characteristics of the birth parameters and of the examined deciduous teeth of the boys aged 5–10 years (N = 56). In the study, one tooth came from one child.

	Total of boys n = 56	Autistic boys n = 22	Healthy boys n = 34	Difference between autistic and healthy boys*
*Birth parameters Median*				
Gestational age (weeks)	39	39.5	39	Z = 0.42; *p* = 0.675
Birth weight (g)	3,400	3,430	3,400	Z = 0.64; *p* = 0.523
Body length (cm)	55	55	55	Z = 0.88; *p* = 0.378
*Primary teeth n (%)*				
Incisors	42 (75.0)	17 (77.3)	25 (73.5)	
Molars	14 (25.0)	5 (22.7)	9 (26.5)	
*Number of accentuated lines n (%)*				
0	34 (60.7)	7 (31.8)	27 (79.4)	
1	8 (14.3)	6 (27.3)	2 (5.9)	
2	8 (14.3)	4 (18.2)	4 (11.8)	
3	4 (7.1)	3 (13.6)	1 (2.9)	
4	2 (3.6)	2 (9.1)	0 (0.0)	
Mean (SD)	0.79 (1.16)	1.41 (1.33)	0.38 (0.82)	
Median	0	1	0	Z = 3.03; *p* = 0.002
*Neonatal line (µm)*				
*n*	56	22	34	
Mean (SD)	14.13 (3.77)	13.98 (3.33)	14.24 (4.07)	
Median	13.64	13.80	13.40	Z = 0.05; *p* = 0.960
*Periodicity of striae of Retzius (days)*				
*n*	21	9	12	
Mean (SD)	10.23 (1.27)	9.75 (1.13)	10.59 (1.29)	
Median	9.64	10.15	10.91	Z = 1.35; *p* = 0.177

*The Mann–Whitney U test.

**Figure 3:**
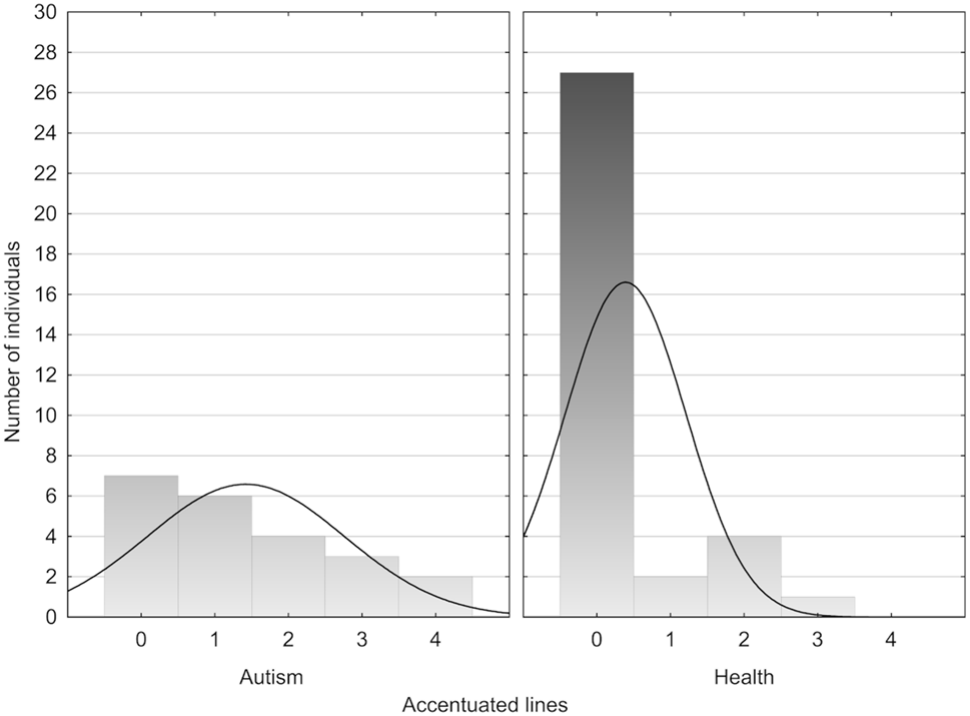
Number of individuals with visible accentuated lines in enamel of primary teeth.

The largest number of accentuated lines (3 and 4) was observed in 3 autistic children born at term (in the 39th, the 40th and the 41st week of gestation) with body weight from 3,300 g to 4,450 g and with body length from 52 to 59 cm). In the control group, 79.4% of the subjects did not exhibit any accentuated lines, and only one child (2.9%) had 3 lines. In contrast, in the autistic group, the absence of accentuated lines was found for 31.8% of the subjects, while two and more lines were identified in 9 children, which accounts for over 40% of this group (Fig. 3).

All the observed accentuated lines both in the autistic children and in the control group were found in postnatal enamel.

The analysis of the width of the neonatal line (NNL) demonstrated a lack of differentiation of this trait between autistic children and healthy children (Z = 0.05; *p* = 0.96) (Table 1). In the case of the boys in the control group, the values of the width of the NNL fluctuated between 9.12 µm and 27.0 µm, with a mean value of 14.13 µm. In the case of the boys diagnosed with autism, the values equaled 9.66 µm, 22.90 µm and 13.98 µm, respectively.

**Figure 4:**
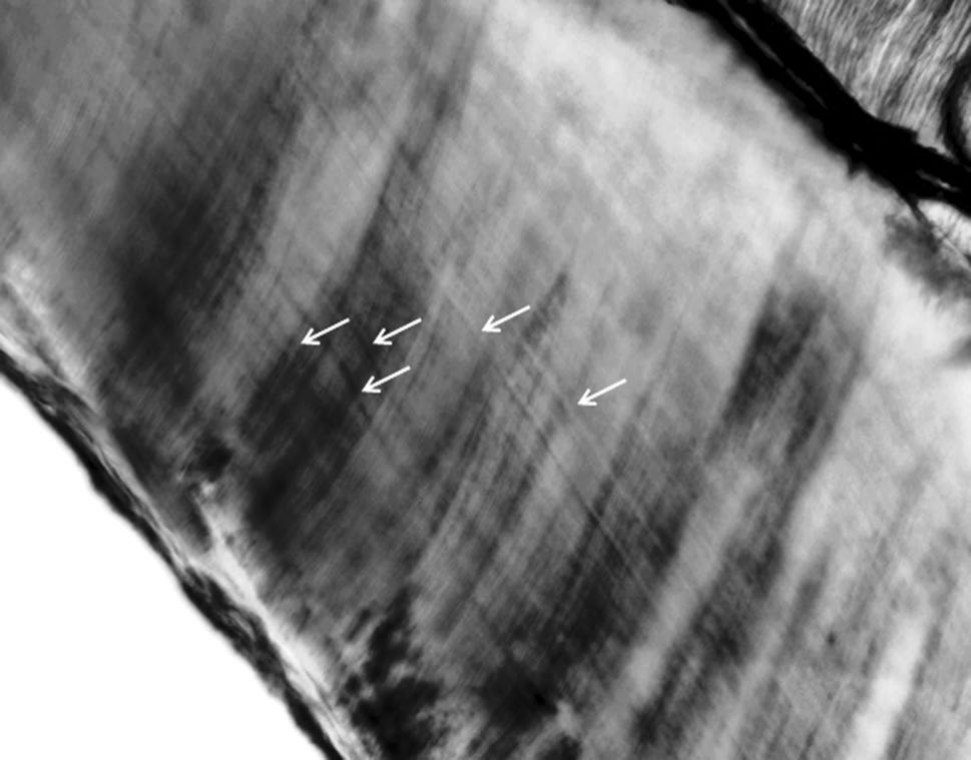
The Retzius lines (white arrows) in cusp of molar (magnification 40x).

In the case of the control group, the distances between adjacent incremental lines could be assessed for ten medial incisors and for two mandibular first molars. In the case of the autistic boys, measurements were performed for six medial incisors, two maxillary first molars and one mandibular first molar. The analysis of the number of days between adjacent striae of Retzius did not demonstrate any differences between autistic children and healthy children (Z = 1.35; *p* = 0.177) (Table 1). Detailed characteristics of the number of accentuated lines, of the width of the NNL and of the periodicity of the striae of Retzius depending on the tooth type in both groups of children is presented in Table 2. Retzius lines visible in molar cusp are presented in Fig. 4.

**Table 2:** Characteristics of the number of accentuated lines, of the width of the neonatal line (NNL) and of the periodicity of the striae of Retzius depending on the tooth type.

	Number of accentuated lines			NNL (µm)			Periodicity of striae of Retzius (days)		
	n	mean	SD	n	mean	SD	n	mean	SD
Total	56	0.79	1.16	56	14.13	3.77	21	10.23	1.26
incisors	42	0.71	1.17	42	14.49	3.95	16	10.27	1.37
i^1^	30	0.83	1.26	30	13.55	3.21	16	10.27	1.37
i^2^	7	0.00	0.00	7	16.48	5.67	–	–	–
i_2_	5	1.00	1.22	5	17.34	3.69	–	–	–
molars	14	1.00	1.11	14	13.06	3.01	5	10.09	1.00
m^1^	5	0.80	1.30	5	13.22	2.72	2	9.76	0.54
m^2^	1	2.00	–	1	12.00	–	–	–	–
m_1_	3	1.33	1.15	3	11.71	1.86	3	10.31	1.30
m_2_	5	0.80	1.10	5	13.91	4.21	–	–	–
Autistic boys	22	1.41	1.33	22	13.98	3.33	9	9.75	1.13
incisors	17	1.65	1.37	17	14.17	3.54	6	9.89	1.34
i^1^	15	1.67	1.35	15	13.94	3.59	6	9.89	1.34
i^2^	–	–	–	–	–	–	–	–	–
i_2_	2	1.50	2.12	2	15.95	3.61	–	–	–
molars	5	0.60	0.89	5	13.30	2.71	3		
m^1^	3	0.33	0.58	3	14.43	2.71	2	9.77	0.54
m^2^	–	–	–	–	–	–	–	–	–
m_1_	1	2.00	–	1	10.00	–	1	8.87	–
m_2_	1	0.00	–	1	13.20	–	–	–	–
Healthy boys	34	0.38	0.82	34	14.24	4.07	12	10.59	1.29
incisors	25	0.08	0.28	25	14.71	4.27	10	10.50	1.39
i^1^	15	0.00	0.00	15	13.17	2.85	10	10.50	1.39
i^2^	7	0.00	0.00	7	16.48	5.67	–	–	–
i_2_	3	0.67	0.58	3	18.27	4.19	–	–	–
molars	9	1.22	1.20	9	12.92	3.31	2	11.03	0.54
m^1^	2	1.50	2.12	2	11.41	1.97	–	–	–
m^2^	1	2.00	–	1	12.00	–	–	–	–
m_1_	2	1.00	1.41	2	12.56	1.60	2	11.03	0.54
m_2_	4	1.00	1.15	4	14.09	4.84	–	–	–

**Figure 5:**
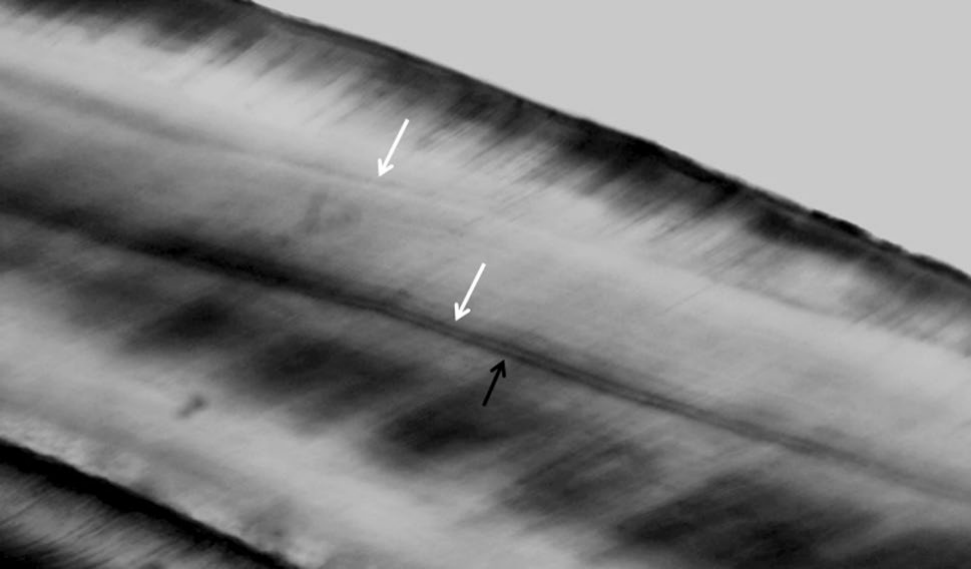
Accentuated lines (white arrows) in enamel of incisor. Note the neonatal line nearby (black arrow) (magnification 40x).

Table 3 presents all the boys in whom accentuated lines were observed in the tooth enamel together with the calculated approximate time (number of days of life) of occurrence of the factor that caused the disturbance in the tooth enamel. A further analysis of the associations of the time of occurrence of disturbances in different tooth types is not possible due to the small size of the sample. An accentuated line nearby NNL is presented in Fig. 5.

**Table 3:** Mean approximate time (number of days of life) of occurrence of the factor that caused the disturbance in the tooth enamel.

Tooth type	Accentuated line	Autistic boys n = 15			Healthy boys n = 7		
		n	Mean	SD	n	Mean	SD
i^1^	1	12	38.5	19.5	–	–	–
	2	7	60.3	17.8	–	–	–
	3	4	79.0	7.3	–	–	–
	4	2	90.0	12.7	–	–	–
i_2_	1	1	54.0	–	2	72.0	9.9
	2	1	69.0	–	–	–	–
	3	1	83.0	–	–	–	–
	4	1	100.0	–	–	–	–
m^1^	1	1	107.0	–	1	43.0	–
	2	–	–	–	1	69.0	–
	3	–	–	–	1	120.0	–
	4	–	–	–	–	–	–
m^2^	1	–	–	–	1	51.0	–
	2	–	–	–	1	105.0	–
	3	–	–	–	–	–	–
	4	–	–	–	–	–	–
m_1_	1	1	59.0	–	1	36.0	–
	2	1	90.0	–	1	87.0	–
	3	–	–	–	–	–	–
	4	–	–	–	–	–	–
m_2_	1	–	–	–	2	61.0	5.7
	2	–	–	–	2	132.0	17.0
	3	–	–	–	–	–	–
	4	–	–	–	–	–	–

## Discussion

Accentuated lines are distinct structures that appear in a developing tooth. They can be observed both in the enamel and in the dentin, and they are connected with a stress factor that caused a metabolic disturbance. The term “accentuated lines” covers both regularly spaced “striae of Retzius exhibiting abnormal prism bending and absence of distortion of prism structure”, also known as “Wilson bands” or “cluster bands”^39^, and marked, accentuated lines which can be superimposed over the rhythmic pattern^7^. Among stress factors which also leave a trace in the form of accentuated lines are mentioned: the mother’s diseases during pregnancy, immunization/vaccination in the first year of life^15^, low birth weight and preterm birth^40–42^ or resource seasonality or periods of drought observed in the case of other primates^24,26^, as well as undernourishment or dietary transitions such as weaning stress^25^. The study by^23^ provides strong evidence that there is an association between physiological stress and the above-mentioned lines. In the study, medical data were used concerning the incidence of such conditions as infections and colds or a treatment with antibiotics in childhood in 19 children. The analysis demonstrated that in the case of a lack of data about an illness, 9 out of 10 children did not have any accentuated lines. In the case of children who had at least one accentuated line, in 8 out of 9 children, conditions or incidents were observed that could indicate the effect of physiological stress.

This study shows that accentuated lines occur in the primary enamel of autistic children significantly more often than in that of healthy children. More accentuated lines in the enamel of primary teeth may indicate a larger number of stressful events experienced by these children or their higher susceptibility to stress factors in the most vulnerable period of life.

Similar results have been reported from studies on the occurrence of accentuated lines in the primary dental enamel of children with Down syndrome, who are also characterized by developmental disturbances and by greater ecosensitivity in the early stages of ontogenesis^43^. Statistically significant differences in the number of accentuated lines in the enamel of primary second molars were also observed in children suffering from developmental disturbances caused by genetic defects—familial dysautonomia syndrome (hereditary sensory and autonomic neuropathy)^27^. All the observed sick children were characterized by the presence of postnatal traumatic lines in the tooth enamel as compared with only 11% in the controls, and the number of the observed disturbances was also significantly larger (3–10 lines in the case of the sick children as compared with a single line in the group of the healthy children).

What can also point to stress factors as a basis for the larger number of accentuated lines in autistic children is the fact that both groups of children do not differ with respect to the parameters of the other lines in the tooth enamel: the neonatal line emerging in the perinatal period and the periodicity of the striae of Retzius attesting the physiological rate of enamel development. The obtained results concerning the width of the NNL in both analyzed groups do not differ from those obtained by^29^ with a mean value of 14.8 μm and by^30^ with a mean value of 10.35 μm. The approximate number of days between adjacent striae of Retzius is also consistent with the period of 6 to 12 days presented in the literature^44–46^.

Research into autism suggests a multifactorial etiology linked to genetic defects and environmental factors affecting the development of the central nervous system, and especially the brain^47–49^. In studies, the issue is also raised that autistic children are characterized by immune dysfunction, which makes them more susceptible to pathogens causing fevers^50,51^. Indeed^52^, reported that autistic children more often contract infections. Viral and bacterial diseases have also been closely associated with developmental defects of tooth enamel. Cytomegalovirus may adversely affect amelogenin and enamelin proteins, cell proliferation and the secretory function of ameloblasts leading to enamel hypoplasia and agenesis^53^. Diseases such as chickenpox, measles, mumps, scarlet fever, and pneumonia may also increase the frequency of enamel hypoplasia^54^. High temperature during an illness may also cause disruptions in enamel development, manifested in the presence of accentuated lines^55,56^. A study conducted on mice and rats confirmed a significant effect of elevated temperature on the functioning of ameloblasts, leading to decreased crown height and enamel hypomineralization^57,58^. Chickenpox and fever are considered factors causing enamel hypomineralization^59,60^.

Researchers increasingly point often to the role of oxidative stress in increasing the risk and clinical manifestations of autism^61^. It has been shown that autistic children exhibit lower levels of glutathione and higher levels of oxidized glutathione^62^, which has been associated with neuronal susceptibility to oxidative stress^63^. Glutathione is the most critical endogenous antioxidant and detoxifier that contributes to reducing environment influence on a variety of cellular processes^64^. Some authors have drawn attention to the relationship between maternal exposure to stress during pregnancy and the risk of autism. For instance^65^ and^66^, found that the mothers of children with this condition experienced significantly more stressful life events than the mothers of healthy offspring. In their study of women exposed to hurricanes and severe tropical storms^67^, identified a strong correlation between those factors and the incidence of autism. In the case of storm exposure, the risk of autism was additionally intensified during gestation months 5–6 and 9–10 as compared with the other months.

Prenatal stress can produce broad effects in postnatal life. Studies on rats show a relationship between such stress and behaviors characteristic of autism, which are probably attributable to disturbances in brain development^68^. Prenatal stress has been found to result in elevated sensitivity and abnormal development of the dopaminergic system, as well as a variety of behavioral abnormalities involving attention, learning, and language^69–72^. Maternal exposure to a prolonged period of stress or repeated stressful episodes may give rise to lifelong hyperactivation of the hypothalamic–pituitary–adrenal (HPA) axis and elevated stress hormone levels^73,74^ reported hypersecretion of cortisol during the day. Also^75^ found that children with autism are characterized by higher levels of salivary cortisol in novel non-social situations, in contrast with the control group. Cortisol is responsible for mechanisms of adaptation of the organism to stress and of maintaining homeostasis,it has an anti-inflammatory and immune suppressive effect, and it also inhibits bone formation and delays healing^76^.

Importantly, cortisol could also lead to disruption in dental development, as it increases the level of calcium ions in the blood, making them less available for enamel mineralization. Thus, the administration of corticosteroids to rats resulted in accentuated surface perikymata and increased the spacing of incremental lines^26,77,^ suggested that accentuated lines in wild baboons may be caused by the stress of their mothers, which may modify cortisol levels in maternal milk^78^.

All the accentuated lines were observed in postnatal enamel, which may be connected with the stability of children’s intrauterine development. It is pointed out in the literature that traces of disturbances caused by stress factors seldom originate in the prenatal period^79^.

Different tooth types are characterized by different periods of enamel formation and mineralization, which allows registering disturbances even up to a year after birth. At birth, incisors are formed in 55–60% depending on their type. In the case of medial incisors, enamel formation ends around the 96^th^ day after birth. In the case of lateral incisors, the period is similar and lasts approximately until the 113th day after birth. The beginning of enamel secretion in molars occurs in a similar period as in the case of incisors, but the secretion lasts much longer—approximately until the 190th day after birth in the case of first molars and approximately until the 389th day after birth in the case of second molars^15^. Similar values were presented by^21^ for deciduous mandibular teeth. In the studies, no significant differences were demonstrated in the frequency of occurrence of accentuated lines depending on the tooth type. In the case of maxillary medial incisors, the first accentuated line was formed on average about 40 days after birth. It is difficult to determine the possible source of the stress factor disturbing enamel formation in this period, because the children’s medical records are not accessible. One possibility is the occurrence of vaccine adverse events (fever and inflammation) after mandatory vaccinations (DPT, Hib, Hep B, pneumococcal vaccine), which are administered in Poland about the 6^th^–8^th^ week of life. According to^15^ immunization/vaccination in the first year of life is one of the stress factors leaving accentuated lines in the tooth enamel. It is not the first vaccination of a child—immediately after birth, a vaccine against tuberculosis and hepatitis B is administered—however, during this procedure, as many as 4 preparations are administered, which can strain a child’s immune system. According to statistical data, in the years 2003–2012, fever was most often observed after vaccination against diphtheria, tetanus and whooping cough—DTP. It is one of the vaccines administered in the above-mentioned period^80^.

About a month after childbirth, breastfeeding women often experience lactation problems, which may result in a discontinuation of this way of feeding the infant and in a transition to modified milk. Studies of children living in Łódź indicate that more than 18% of women who start breastfeeding after giving birth continue to breastfeed for a period shorter than 2 months^81^. Dietary transitions may be connected with stress, which can result in disturbances in the functioning of ameloblasts in a child, although studies confirming the association of a dietary transition with accentuated lines in the tooth enamel were only conducted on primates^27^.

To the best of our knowledge, there are no literature reports concerning the development of primary dental enamel in autistic children. Most studies focus on assessing the levels of the organic chemicals relevant to autism etiology detected in primary teeth, and also of such metals as mercury, lead, manganese, zinc and copper^82–86^. However, there are many publications analyzing the relationship between enamel structure and prenatal and perinatal factors, e.g.^17,22,27,29,30,87^ as well as^88^, who stated after a precise meta-analysis that teeth are potential new tools to measure early-life biological stress and subsequent mental health risk.

The present study has identified a higher frequency of accentuated lines in the enamel of primary teeth of autistic children, which may be a sign of stress factors in the first years of life or may indicate a higher susceptibility of children with this disorder to environmental factors. Further analyses should be conducted on more extensive odontological material from different populations including an analysis of medical history concerning pregnancy and the first years of the child’s life.
